# Emerging robotic platforms in partial nephrectomy: a comparative systematic review and network meta-analysis

**DOI:** 10.1007/s11701-026-03467-6

**Published:** 2026-05-21

**Authors:** Mohammad N. Almajali, Saba’a Darwish, Ahmad Bani-ata, Leen Alkelani, Adam Eugene Davis, Muhammed Hammad, Ryan W. Dobbs, David I. Lee, Mohammed Shahait

**Affiliations:** 1 Faculty of medicine, Jordan University of Science and Technology, Irbid, 21110 Jordan; 2 Philadelphia College of Osteopathic Medicine-Georgia Campus, Suwannee, GA USA; 3 Urology Department, University of California-Irvine, Orange, CA USA; 4 Urology Department, Cook County Health & Hospitals, Chicago, IL USA

**Keywords:** Robotics, Partial Nephrectomy, Emerging robotic platforms, Perioperative outcomes, Network meta-analysis, Minimally invasive

## Abstract

Robotic-assisted partial nephrectomy (RAPN) is the preferred minimally invasive nephron-sparing technique, with outcomes comparable to open and laparoscopic surgery. While most evidence derives from the da Vinci system, newer robotic platforms such as Hugo, Hinotori, KangDuo and MP1000 are emerging. This systematic review and network meta-analysis evaluated perioperative outcomes across available RAPN platforms. A systematic search of PubMed, Cochrane Library, CINAHL, and Scopus identified studies reporting RAPN outcomes across available robotic platforms. Eligible studies included randomized and observational cohorts. Risk of bias was assessed with ROBINS-I. Outcomes included in the analysis operative time (OT), estimated blood loss (EBL), and length of hospital stay (LOS). A frequentist NMA using the “netmeta” R package generated standardized mean differences (SMD) with 95% CIs, and P-scores ranked platform performance. The review was registered on PROSPERO (ID: CRD420251032233). This review was conducted in accordance with PRISMA guidelines. Thirteen studies (*n* = 2,450) were included. Under fixed-effects model, da Vinci SP (SMD − 0.63, 95% CI − 0.87 to − 0.40) and Xi (–0.52, 95% CI − 0.67 to − 0.38) revealed shorter operative times; however, under random-effects models these differences were not statically significant due to substantial heterogeneity. Heterogeneity (I² ≈ 84%) attenuated these effects. For ischemia time, Hugo RAS (–0.88, 95% CI − 1.45 to − 0.30) and Xi (–0.58, 95% CI − 0.73 to − 0.44) outperformed Si, though differences lost significance under random-effects (I² >90%). No significant differences were found for length of stay, blood loss, or R.E.N.A.L. nephrometry scores, although ranking differences were observed. Preoperative outcomes were revealed a comparable outcome using emerging robotic system with the established system. Favourable trends in operative and ischemia times were showed in some platforms; however, these differences were not vigorous under random-effects models and are limited by substantial heterogeneity. This raised the need for long-term, comparative follow-up studies, as the current evidence remains elementary.

**Supplementary Information:**

The online version contains supplementary material available at 10.1007/s11701-026-03467-6.

## Introduction

The evolution of partial nephrectomy from open surgery to minimally invasive techniques is well established, with laparoscopic and robotic approaches now preferred when oncologic and functional outcomes are not compromised, as recommended by the American Urological Association (AUA) [1]. Following the 2000 FDA approval of the Da Vinci robotic platform, robotic-assisted partial nephrectomy (RAPN) was rapidly adopted and has since become the predominant minimally invasive nephron-sparing approach [2].

The AUA now considers minimally invasive techniques, including RAPN, a standard of care for appropriately selected patients, offering oncologic and functional outcomes equivalent to those of open surgery [1]. Multiple recent network meta-analyses and large multicenter studies demonstrate that RAPN is associated with reduced estimated blood loss, shorter hospital stay, and lower complication rates compared to both open and laparoscopic approaches [2–6].

Evidence also supports improved functional preservation with RAPN, including lower rates of acute kidney injury and new-onset chronic kidney disease following surgery, compared to open partial nephrectomy [7–9]. While new robotic platforms such as Hugo, Hinotori, Revo-i, and Senhance have emerged, comparative outcome data across platforms remain limited. Most existing meta-analyses focus exclusively on the Da Vinci system, with no comprehensive network meta-analysis currently available to assess RAPN outcomes across multiple robotic platforms [2–4].

Despite the widespread of new robotics platforms, a persistent comparative limitation of outcome data across systems still present. To date, no comprehensive network meta-analysis has compared perioperative outcomes across multiple robotic platforms used RAPN. This study aims to fill the gap by comparing perioperative outcomes among all available robotic platforms, addressing a significant limitation in the current body of evidence.

## Methods

### Study design

This systematic review and network meta-analysis adhered to the Cochrane Handbook of Systematic Reviews of Interventions [10] and the PRISMA-NMA guidelines [11]. The analysis was based on a pre-registered protocol (CRD420251032233) listed in the PROSPERO database. Ethical and Institutional Review Board approval was not required because the research exclusively utilized publicly accessible secondary data from previously published studies.

### Eligibility criteria

The review focused on studies involving adult patients who underwent Robot-Assisted Partial Nephrectomy (RAPN) that were published from 2000 to present. Only studies directly comparing two or more of the specified robotic surgical platforms (“Da Vinci Si”, “Da Vinci SP”, “Da Vinci Xi”, “Kang Duo”, “Hugo RAS”, “MP1000”) were included. Eligible studies need to report on perioperative outcomes (e.g. operative time, estimated blood loss, complications, conversion rates, ischemia time, etc.), oncological outcomes (e.g. surgical margins) and/or survival outcomes. Cohorts, Randomized Controlled Trials (RCTs), Non-Randomized Controlled Trials (Non-RCTs) and Case Series with more than > 3 cases were included. Exclusions comprised studies with overlapping patient data, case reports, animal studies, non-English papers, surveys, letters to editors, book chapter, non-peer-reviewed articles and case series with 3 or fewer cases.

### Screening and selection process

A systematic literature search was conducted in the Cochrane Library, CINAHL, PubMed, and Scopus databases, as well as relevant trial registries, on February 26, 2025. The search strategy included the terms “minimally invasive partial nephrectomy,” “robotic-assisted partial nephrectomy,” “RAPN,” and the names of specific robotic platforms (detailed in Supplementary Table 1).

Two reviewers (SD and LA) independently screened study titles, abstracts, and full texts using Covidence. Discrepancies were resolved through discussion or consultation with a third reviewer (MA).

The search identified a total of 599 records. After removal of 76 duplicates (61 identified using Covidence and 15 manually), 523 records remained for title and abstract screening, of which 387 were excluded for not meeting eligibility criteria.

A total of 136 full-text articles were assessed for eligibility. Of these, 52 studies were excluded for the following reasons: non-English language (*n* = 10), animal studies (*n* = 3), cadaveric studies (*n* = 1), book chapters (*n* = 3), wrong comparator (*n* = 9), wrong study design (*n* = 9), wrong intervention (*n* = 10), wrong outcomes (*n* = 2), combined outcomes (*n* = 2), combined surgeries (*n* = 1), wrong indication (*n* = 1), and case series with three or fewer patients (*n* = 1).

**Fig. 1:**
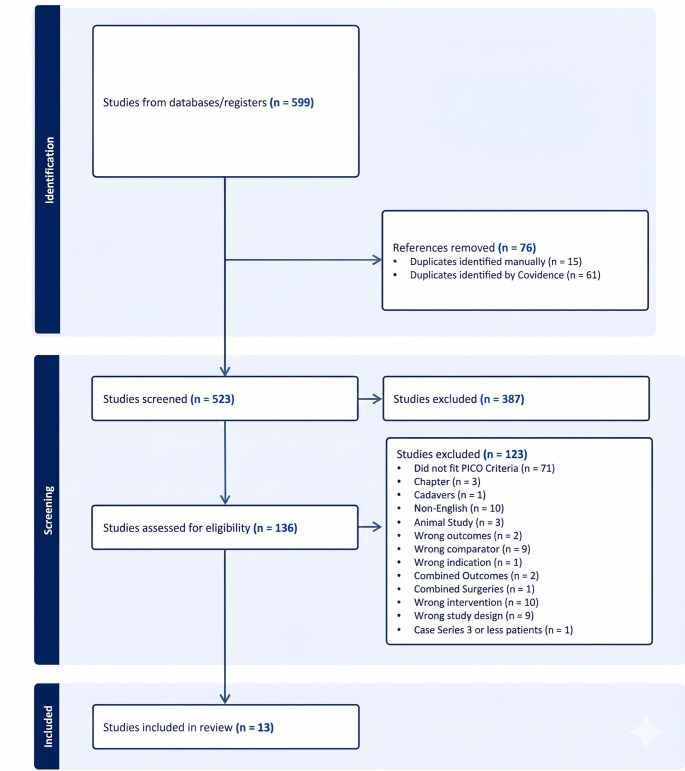
The PRISMA flow diagram depicting the selection process.

Ultimately, 13 studies met the inclusion criteria and were included in the quantitative synthesis. The study selection process is illustrated in the PRISMA flow diagram (Fig. 1).

### Extraction of data

To ensure precision and uniformity, three reviewers (SD, AA, LA) independently extracted data using a standardized template. Extracted information encompassed study characteristics (authors, publication year, study design and sample size), patients’ demographics (age, BMI, Underlying causes, comorbidities), intervention specifics (surgical procedure, platform) and the outcomes of interest: perioperative outcomes (operative times, estimated blood loss, ischemia time, length of hospital stay) and R.E.N.A.L. Nephrotomy score.

### Risk of bias assessment

The ROBINS-I (Risk of Bias in Non-Randomized Studies of Interventions) tool was employed by two independent reviewers (SD, LA) to evaluate potential bias across seven domains: confounding, participant selection, intervention classification, derivations from intended interventions, missing data, outcome measurement and selection of reported results. Each study received a risk of low, moderate, serious or critical. Disagreements were settled via discussion. The R package robvis was used to concisely visualize the risk assessment results.

### Endpoints

Our primary endpoints were operative time (skin incision to skin closure) and warm ischemia time (clamp-on to clamp-off). Secondary endpoints included estimated blood loss, length of hospital stay and R.E.N.A.L nephrectomy score. We also extracted data on complications, conversion rates and oncologic outcomes whenever studies reported them. However, because definitions and reporting methods varied too widely across studies, we did not pool these additional endpoints statistically. Instead, we summarized them narratively.

### Statistical analysis

A comprehensive frequentist network meta-analysis (NMA) employing graph-theoretical methods was conducted using the netmeta package in R (version 4.5.0). The network structure for each outcome was visualized through geometry plots, depicting robotic platforms as nodes and direct pairwise comparisons as connecting lines. Line thickness corresponded to the number of comparisons and participants involved.

Effect sizes were calculated as Standardized Mean Differences (SMD) with 95% Confidence Intervals (95% CI). Treatment effects were first estimated under a common effects model, followed by a random effects model to account for between-study heterogeneity. Model selection was guided by heterogeneity assessment: a random effects model was applied when significant heterogeneity was present (*p* < 0.05 or I² >50%), while a fixed effects model was used otherwise. Heterogeneity was quantified using the between-study variance (τ²) and I² statistics. Global heterogeneity across the network was evaluated using Cochran’s Q statistic. Global inconsistency (disagreement between direct and indirect evidence) was assessed using the design-by-treatment interaction model. Where applicable, local inconsistency within specific network loops was further investigated using node-splitting analysis.

The relative effectiveness of the robotic platforms was probabilistically ranked using P-scores. This frequentist metric, analogous to the surface using the cumulative ranking curve (SUCRA), ranges from 0 (least effective) to 1 (most effective) for a given outcome (e.g. a higher P-score indicates a greater likelihood of being the most effective platform in reducing blood loss). Forest plots visually presented individual study results alongside pooled estimates, facilitating cross-platform comparisons.

Reporting varied across studies; therefore, we could not perform stratified analyses based on factors such as tumor complexity, surgeon experience or surgical approach.

## Results

### Study selection

The database search identified 599 records. After removal of 76 duplicates, 523 studies underwent title and abstract screening. Of these, 387 were excluded. A total of 136 full-text articles were assessed for eligibility, and 13 studies met inclusion criteria and were included in the quantitative synthesis (Fig. 1).

### Study characteristics

Table 1 summarizes the characteristics of the included studies (*n* = 13), encompassing a total of 2,450 patients across diverse geographic regions including China, Japan, Korea, Taiwan, Lebanon, Egypt, Europe, and the USA. The studies included both randomized controlled trials (RCTs; *n* = 3) and retrospective cohort studies (*n* = 10). Comparisons involved established robotic platforms (Da Vinci Si, Xi and SP) and emerging systems (KangDuo, Hinotori, MP1000, Hugo RAS). Surgical approaches varied, with transperitoneal and retroperitoneal techniques most frequently reported. Rankings of the outcomes can be found in Table 2.

**Table 1:** Characteristics of all studies included in the network meta-analysis

Author, Year	Country	Study design	Comparison	Total	Platform 1 (*N*)	Platform 2 (*N*)	Approach
Li et al., 2024	China	RCT	Da Vinci Si v KangDuo	56	28	28	NA
Gao et al., 2024	China	RCT	Da Vinci Si v MP1000	54	28	26	Transperitoneal, Retroperitoneal
Motoyama et al., 2023	Japan	Cohort	Da Vinci v Hinotori	343	303	40	Transperitoneal, Retroperitoneal
Li et al., 2023	China	RCT	Da Vinci Si v KangDuo	99	50	49	Transperitoneal, Retroperitoneal
Bravi et al., 2023	Belgium	Cohort	Da Vinci Si v Da Vinci Xi	447	184	263	NA
GarcíaRojo et al., 2024	Spain	Cohort	Da Vinci Xi v Hugo RAS	50	25	25	NA
Bang et al., 2024*	Korea	Cohort	Da Vinci SP v Da Vinci Xi	88	44	44	Retroperitoneal
AbdelRaheem et al., 2017	Korea, Egypt	Cohort	Da Vinci Si v Da Vinci Xi	36	18	18	Transperitoneal
El-Asmar et al., 2021	Lebanon	Cohort	Da Vinci Si 3-arm v Da Vinci Si 4-arm	80	40	40	Transperitoneal
Okhawere et al., 2025	USA	Cohort	Da Vinci SP v Da Vinci Xi	286	86	200	Retroperitoneal
Licari et al., 2024*	USA	Cohort	Da Vinci SP v Da Vinci Xi	60	30	30	Transperitoneal, Retroperitoneal
Chen et al., 2023	Taiwan	Cohort	Da Vinci Si v Da Vinci Xi	338	226	112	Transperitoneal, Retroperitoneal
Palacios et al., 2022	USA	cohort	Da Vinci SP v Da Vinci Xi	62	20	42	Retroperitoneal

Abbreviations: RCT – Randomized Controlled Trial, SP – Single Port, MP – Multiple Port

*Propensity Score Matched

**Table 2:** Ranking of robotic platforms for intraoperative, oncological and postoperative outcomes

	1st	2nd	3rd	4th	5th	6th	7th	8th	9th
Length of stay	da vinci SP (0.782)	da vinci xi (0.481)	KangDuo (0.470)	da vinci si (0.439)	Hugo RAS (0.327)				
Operation Time	da vinci SP (0.872)	da vinci xi (0.720)	da vinci si (0.316)	MP1000 (0.092)					
Ischemia time	Hugo RAS (0.791)	da vinci xi (0.708)	da vinci SP (0.589)	da vinci si (0.322)	MP1000 (0.314)	KangDuo (0.276)			
Blood Loss	Hugo RAS (0.944)	da vinci si (0.650)	da vinci xi (0.549)	da vinci SP (0.477)	MP1000 (0.207)	KangDuo (0.174)			
Renal Score	da vinci SP (0.684)	KangDuo (0.483)	da vinci si (0.474)	da vinci xi (0.445)	Hugo RAS (0.413)				

Abbreviations: SP – Single Port, MP – Multiple Port

### Demographic and clinical characteristics of patients

Baseline characteristics were generally comparable across studies (Table 3). Mean patient age ranged from 50 to 65 years, and BMI ranged from 23 to 32 kg/m² depending on geographic region. Most studies included tumors with moderate complexity (R.E.N.A.L. score 7–9), although some SP and KangDuo cohorts reported lower complexity lesions. Tumor size was typically between 23 and 40 mm. Comorbidity burden was modest, with Charlson Comorbidity Index values generally between 2 and 4.

**Table 3:** Patients’ characteristics in all studies included in the network meta-analysis

Author, Year	Platform	Age (years)	BMI (kg/m2)	CCI	Previous Surgeries, *n* (%)	*R*.E.*N*.A.L Score	*P*.A.D.U.A Score	Tumor size (mm)
Li et al., 2024	Da Vinci Si	50.98 ± 13.33	26.28 ± 3.64	NA	7.00 (25.00%)	4-6: 13 (46.43%), 7-9: 15 (53.57%), 10-12: 0	NA	NA
	KangDuo	54.97 ± 10.18	26.05 ± 2.59		10.00 (35.71%)	4-6: 19 (67.86%), 7-9: 9 (32.14%), 10-12: 0		
Gao et al., 2024	Da Vinci Si	56.18 ± 10.36	24.68±2.84	NA	NA	NA	NA	NA
	MP1000	52.12 ± 10.99	24.89±2.87					
Motoyama et al., 2023	Da Vinci	65 (18-90)*	23.9 (14.4-48.7)*	NA	NA	7 (4-11)*	NA	25.00 (5.00-77.00)*
	Hinotori	63 (37-84)*	23.6 (15.3-48.9)*			8 (4-10)*		27.00 (8.00-53.00)*
Li et al., 2023	Da Vinci Si	52.14 ± 12.38	25.63 ± 3.29	NA	15 (30.00%)	NA	NA	NA
	KangDuo	54.36 ± 10.25	25.80 ± 2.84		22 (44.90%)			
Bravi et al., 2023	Da Vinci Si	62 (53-72)	27 (25-27)	NA	NA	NA	6: 17 (9%), 7-9: 112 (61%), 10+: 55 (30%)	30.00 (20.00-40.00)
	Da Vinci Xi	65 (55-73)	27 (26-27)				6: 34 (13%), 7-9: 167 (63%), 10+: 62 (24%)	30.00 (30.00-30.00)
GarcíaRojo et al., 2024	Da Vinci Xi	63.56 ± 9.31	26.5 ± 4.11	3.36 ± 1.44	NA	5.60 ± 2.12	7.00 ± 2.08	32.90 ± 11.66
	Hugo RAS	61.48 ± 9.71	27.9 ± 3.89	3.92 ± 1.15		5.76 ± 2.81	6.88 ± 3.01	35.00 ± 21.30
Bang et al., 2024	Da Vinci SP	53.73 ± 12.07	25.29 ± 3.07	NA	NA	4.73 ± 0.97	NA	23.50 ± 11.70
	Da Vinci Xi	52.89 ± 13.45	25.29 ± 3.07			4.70 ± 0.93		29.40 ± 10.40
AbdelRaheem et al., 2017	Da Vinci Si	50.30 ± 9.20	25.8 ± 3.4	NA	NA	NA	6-7: 3 (16.7%), 8-9: 6 (33.3%), 10+: 9 (50%)	C: 40.00 ± 16.00 P: 35.00 ± 17.00
	Da Vinci Xi	50.90 ± 8.20	24.3 ± 3.7				6-7: 5 (27.8%), 8-9: 5 (27.8%), 10+: 8 (44.4%)	C: 36.00 ± 18.00 P: 31.00 ± 19.00
El-Asmar et al., 2021	Da Vinci Si 3-arm	NA	NA	NA	NA	4-6: 23 (57.5%), 7-9: 15 (37.5%), 10-12: 2 (5%)	NA	31.40 ± 11.90
	Da Vinci Si 4-arm					4-6: 13 (36.1%), 7-9: 21 (58.3%), 10-12: 2 (5.6%)		34.40 ± 16.00
Okhawere et al., 2025	Da Vinci SP	60.00 ± 12.00	30.00 ± 6.00	3 (2-4)	NA	5 (4-7)	NA	27.00 ± 13.20
	Da Vinci Xi	62.00 ± 13.00	30.00 ± 7.00	3 (2-5)		8 (6-9)		29.60 ± 13.30
Licari et al., 2024	Da Vinci SP	63 (55.2-67.8)	29.40 (25.10-32.60)	2 (1–3)	12 (40.00%)	5 (4-7)	NA	31.00 (22.00-40.00)
	Da Vinci Xi	58 (52-66)	32.60 (27.70-37.10)	2 (1–3)	11 (37.00%)	5 (4-7)		35.00 (22.00-49.00)
Chen et al., 2023	Da Vinci Si	55 (46-66)	25 (22.3-27.2)	NA	NA	7 (6-8)	NA	34.00 (26.00-46.00)
	Da Vinci Xi	56 (47-66)	25 (23.2-27.4)			7 (5-8)		32.00 (24.00-44.00)
Palacios et al., 2022	Da Vinci SP	56.5 (48.5-65)	30.75 (25.2–38.8)	4.5 (2.5-6)	NA	4 (4-6)	NA	30.00 (24.00-42.00)
	Da Vinci Xi	60 (54-64)	29.88 (25.1–33.1)	3.5 (2-5)		6 (5-7)		28.00 (24.00-36.00)

SP–Single Port, MP–Multiple Port, BMI–Body Mass Index, CCI–Charlson Comorbidity Index, C–Clinical, P–Pathological

Continuous variables presented as mean ± SD or median (interquartile range) unless specified otherwise

*Median (Range)

### Surgical outcomes and perioperative metrics

Table 4 represents operative outcomes demonstrated variation between robotic platforms. Operative time ranged from 89 to 225 min for Da Vinci Si, 112–201 min for Xi, 95–166 min for SP, ~ 170 min for Hinotori, and 124 min for MP1000. The shortest times were reported in Chinese cohorts with Da Vinci Si (median 89 min), while the longest were observed in Western SP and Xi series (up to 189 min). Warm ischemia time ranged between 13 and 22 min. The shortest times were reported for Hinotori (~ 12 min), while longer durations were noted in SP (~ 22 min). Da Vinci Si and Xi reported values of 16–19 min, and KangDuo showed slightly longer clamp times (~ 18 min).

**Table 4:** Surgery Characteristics for all studies included in the network meta-analysis

Author, Year	Platform	Operative time (mins)	Ischemia time (mins)	Estimated blood loss (ml)	Length of stay (days)	Positive margin, *n* (%)
Li et al., 2024	Da Vinci Si	89.00 (77.00–103.75)	16.85 ± 5.70	20.00 (10.00–45.00)	4.00 (4.00–4.00)	0.00 (0.00%)
	KangDuo	112.50 (94.25–143.75)	17.97 ± 4.70	10.00 (2.75–20.00)	4.00 (4.00–4.00)	0.00 (0.00%)
Gao et al., 2024	Da Vinci Si	100.00 (81.00–135.00)	16.00 (11.61–24.00)	25.00 (20.00–50.00)	NA	0.00 (0.00%)
	MP1000	124.50 (100.00–155.00)	18.00 (12.25–24.00)	50.00 (20.00–100.00)		0.00 (0.00%)
Motoyama et al., 2023	Da Vinci	170 (97–446)*	13 (4–44)*	50 (0-4195)*	8 (4-31)*	2 (0.70%)
	Hinotori	170 (106–268)*	12 (5–20)*	35 (1-312)*	6 (4-23)*	0.00 (0.00%)
Li et al., 2023	Da Vinci Si	NA	17.00 ± 6.03	30 (20-50)	4 (4-8)*	0.00 (0.00%)
	KangDuo		18.38 ± 5.48	50 (10-50)	4 (3-10)*	0.00 (0.00%)
Bravi et al., 2023	Da Vinci Si	140 (120–180)	13 (10–17)	NA	5 (4-5)	5 (3.40%)
	Da Vinci Xi	170 (124–205)	14 (11–20)		3 (3-4)	14 (6.50%)
GarcíaRojo et al., 2024	Da Vinci Xi	NA	14.38 ± 15.61	233.80 ± 265.75	2.24 ± 0.60	1 (4.00%)
	Hugo RAS		9.92 ± 11.94	142.40 ± 85.25	2.40 ± 0.65	2 (8.00%)
Bang et al., 2024	Da Vinci SP	95.43 ± 32.22	13.82 ± 4.59	92.27 ± 104.30	4.52 ± 2.50	NA
	Da Vinci Xi	103.68 ± 21.89	17.18 ± 6.56	90.91 ± 91.06	4.07 ± 0.59	
AbdelRaheem et al., 2017	Da Vinci Si	158.20 ± 43.80	21.50 ± 11.30	330.60 ± 194.80	5.60 ± 1.50	0.00 (0.00%)
	Da Vinci Xi	155.10 ± 37.40	17.00 ± 10.60	331.10 ± 176.20	5.70 ± 1.50	0.00 (0.00%)
El-Asmar et al., 2021	Da Vinci Si 3-arm	NA	17.40 ± 6.16	247.50 ± 135.38	3.98 ± 1.05	2 (5.10%)
	Da Vinci Si 4-arm		17.15 ± 5.35	244.36 ± 239.02	4.25 ± 1.79	3 (7.50%)
Okhawere et al., 2025	Da Vinci SP	130.00 ± 45.00	22.00 ± 8.00	109.00 ± 196.00	1.00 ± 1.98	1 (2.30%)
	Da Vinci Xi	133.00 ± 87.00	16.00 ± 7.00	94.00 ± 122.00	1.15 ± 0.72	8 (4.00%)
Licari et al., 2024	Da Vinci SP	155.5 (126–241.2)	25 (20–37)	50 (50–237.5)	1.04 (1–1.44)	5 (16.60%)
	Da Vinci Xi	189.5 (170.2–229)	27 (21–32)	100 (50–200)	1.42 (1.26–2)	4 (13.30%)
Chen et al., 2023	Da Vinci Si	225 (188–257)	28 (20–38)	150 (75–300)	6 (6–8)	42 (18.60%)
	Da Vinci Xi	201 (165–244)	19 (12–25)	200 (50–588)	7 (6–7)	29 (25.90%)
Palacios et al., 2022	Da Vinci SP	166.5 (135.5–201)	25 (17–27)	50 (20–50)	1 (1–1)	0.00 (0.00%)
	Da Vinci Xi	158.5 (143–193)	20 (16–25)	50 (25–100)	2 (1–3)	4 (10.00%)

SP–Single Port, MP–Multiple Port

Continuous variables presented as mean ± SD or median (interquartile range) unless specified otherwise

*Median (Range)

Estimated blood loss (EBL) ranged from ~ 90–100 ml in Asian SP and Xi cohorts to 100–200 ml in Western cohorts. Length of stay (LOS) differed by region. Asian studies reported 3–6 days, while Western Xi and SP cohorts discharged patients within 1–2 days. Positive surgical margins (PSM) were reported at low rates across most series (0–7.5%). Higher rates were recorded in larger cohorts, such as in Chen et al., with 18.6% for Da Vinci Si and 25.9% for Da Vinci Xi.

### Networking meta-analysis results

#### Length of hospital stay

**Fig. 2:**
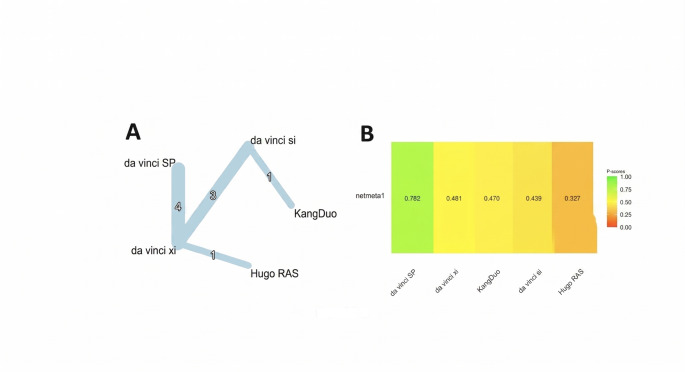
(**A**) Graph network meta-analysis for the effect of robotic surgical platforms on postoperative length of stay (LOS). (**B**) Bar plot showing the platform ranking on LOS

A frequentist, graph-theoretical network meta-analysis was conducted to compare the effect of five robotic surgical platforms on postoperative length of stay (LOS): da Vinci Si (reference), da Vinci SP, da Vinci Xi, Hugo RAS, and KangDuo. The analysis included 9 studies, comprising 9 pairwise comparisons across 4 distinct study designs (Fig. 2A).

Under both the common-effects and random-effects models, no statistically significant differences in LOS were observed between any of the alternative robotic platforms and da Vinci Si. Between-study heterogeneity was estimated as τ² = 0.036 (τ = 0.19), corresponding to moderate heterogeneity with an I² of 55% (95% CI: 0% to 82%).

Under the common-effects model, the da Vinci SP system ranked highest (P-score = 0.86), indicating the greatest likelihood of being associated with the shortest LOS. This was followed by da Vinci Xi (0.55), KangDuo (0.43), da Vinci Si (0.35), and Hugo RAS (0.31).

When allowing for between-study heterogeneity via the random-effects model, the absolute P-scores decreased slightly, yet the general hierarchy at the extremes was preserved: da Vinci SP remained the top-ranking system (0.78), while Hugo RAS continued to rank lowest (0.33). The middle positions showed slight convergence—Xi (0.48) and KangDuo (0.47) became nearly indistinguishable, and Si modestly improved to 0.44 (Fig. 2B). These shifts reflect the added uncertainty introduced by the moderate level of heterogeneity observed in the network (τ ≈ 0.19).

#### Operation time

**Fig. 3:**
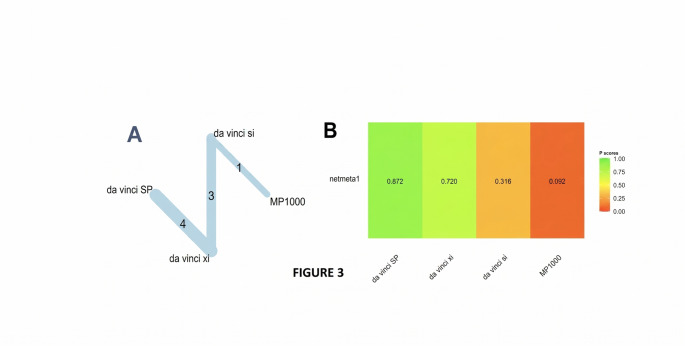
(**A**) Graph network meta-analysis for the effect of robotic surgical platforms on operation time. (**B**) Bar plot showing the platform ranking on operation time

A graph-theoretical network meta-analysis combined data from 8 studies evaluating four robotic surgical systems—da Vinci Si (reference), da Vinci SP, da Vinci Xi, and MP1000—across 8 direct comparisons and 3 study designs (Fig. 3A).

Under the common-effects model, both next-generation da Vinci platforms were associated with statistically significant and moderate reductions in operation time compared to the legacy Si system: da Vinci SP (SMD = − 0.63, 95% CI: − 0.87 to − 0.40; *p* < 0.0001) and da Vinci Xi (SMD = − 0.52, 95% CI: − 0.67 to − 0.38; *p* < 0.0001). MP1000 demonstrated a longer point estimate for operative time; however, the confidence interval crossed the null and the difference was not statistically significant.(SMD = + 0.46, 95% CI: − 0.08 to + 1.00; *p* = 0.10).

When allowing for between-study heterogeneity using the random-effects model, the estimated benefits for SP and Xi were attenuated and became statistically non-significant: SP (SMD = − 0.53, 95% CI: − 1.19 to + 0.14; *p* = 0.12) and Xi (SMD = − 0.39, 95% CI: − 0.89 to + 0.10; *p* = 0.12). The result for MP1000 remained non-significant (*p* = 0.34). The analysis revealed substantial heterogeneity across included studies (τ² = 0.16; τ = 0.40), corresponding to I² ≈ 84% (95% CI: 67% to 92.

Under the common-effects model, the da Vinci SP platform was most strongly favored (P-score = 0.96), indicating the highest probability of achieving the shortest operation time. Da Vinci Xi followed with a P-score of 0.71, while the older da Vinci Si platform ranked considerably lower (0.32). The investigational MP1000 system ranked last (0.02), reflecting a tendency toward longer operative durations.

When accounting for substantial between-study heterogeneity (τ ≈ 0.40) using a random-effects model, the overall P-scores declined but the relative hierarchy remained intact: SP maintained its lead (0.87), MP1000 continued to rank lowest (0.09), and Xi preserved a strong second-place position (0.72). The Si platform remained in the middle tier (0.32), consistent with its legacy status (Fig. 3B).

#### Ischemia time

**Fig. 4:**
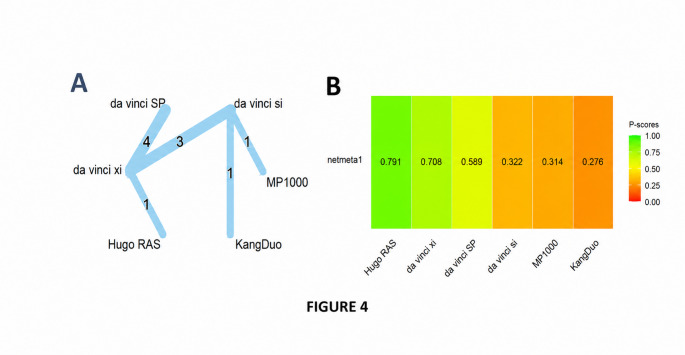
(**A**) Graph network meta-analysis for the effect of robotic surgical platforms on ischemia time. (**B**) Bar plot showing the platform ranking on ischemia time

A graph-theoretical network meta-analysis synthesized data from 10 randomized and observational studies evaluating six robotic surgical platforms—da Vinci Si (reference), da Vinci SP, da Vinci Xi, Hugo RAS, KangDuo, and MP1000—across 10 direct comparisons and 5 distinct study designs (Fig. 4A).

Under the common-effects model, statistically significant reductions in ischemia time were observed for da Vinci Xi (SMD = − 0.58; 95% CI: − 0.73 to − 0.44; *p* < 0.0001) and Hugo RAS (–0.88; 95% CI: − 1.45 to − 0.30; *p* = 0.0028), suggesting improved performance relative to da Vinci Si. da Vinci SP showed a numerically shorter ischemia time(–0.24; 95% CI: − 0.48 to 0.00; *p* = 0.051), but this did not achieve conventional statistical significance, while KangDuo and MP1000 demonstrated non-significant, slightly positive effects, indicating modestly longer ischemia times compared with the reference.

By contrast, the random-effects model—which accounts for between-study variability—found that none of the platforms differed significantly from da Vinci Si. Point estimates remained in the same direction but were accompanied by wide confidence intervals that included the null: da Vinci Xi (–0.50; 95% CI: − 1.17 to 0.18; *p* = 0.149), Hugo RAS (–0.80; 95% CI: − 2.21 to 0.61; *p* = 0.262), and da Vinci SP (–0.38; 95% CI: − 1.28 to 0.52; *p* = 0.407). Assessment of between-study heterogeneity revealed substantial dispersion (τ² = 0.317; I² = 91.3%, 95% CI: 83.8%–95.3%).

Under the common-effects model, Hugo RAS emerges as the leading platform (P score ≈ 0.97), followed by da Vinci Xi (0.83) and da Vinci SP (0.57). The legacy da Vinci Si and the investigational platforms MP1000 and KangDuo rank markedly lower, with scores of 0.30, 0.21, and 0.13, respectively, reflecting a lower probability of achieving the shortest ischemia time.

When accounting for substantial between-study heterogeneity in the random-effects model, the absolute P scores decrease and their spread narrows, yet the relative ordering remains largely preserved: Hugo RAS (0.79) maintains its lead, followed by da Vinci Xi (0.71), while MP1000 (0.31) and KangDuo (0.28) continue to occupy the bottom tier; da Vinci Si (0.32) remains intermediate (Fig. 4B).

#### Blood loss

**Fig. 5:**
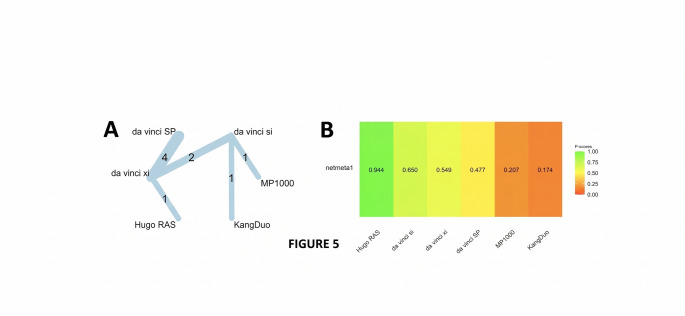
(**A**) Graph network meta-analysis for the effect of robotic surgical platforms on blood loss. (**B**) Bar plot showing the platform ranking on blood loss

A graph-theoretical network meta-analysis synthesized data from 9 randomized and observational trials evaluating six robotic surgical platforms—da Vinci Si (reference), da Vinci SP, da Vinci Xi, Hugo RAS, KangDuo, and MP1000—across 9 direct comparisons and 5 distinct study designs (Fig. 5A).

Under both the common-effects model and the random-effects model (which converged due to zero estimated heterogeneity, τ² = 0), none of the platforms demonstrated a statistically significant difference in blood loss compared with da Vinci Si. All point estimates were small, and their 95% confidence intervals included the null value: da Vinci SP (SMD = + 0.07; 95% CI: − 0.21 to + 0.35; *p* = 0.63), da Vinci Xi (+ 0.04; 95% CI: − 0.17 to + 0.26; *p* = 0.70), KangDuo (+ 0.32; 95% CI: − 0.08 to + 0.71; *p* = 0.12), MP1000 (+ 0.32; 95% CI: − 0.22 to + 0.86; *p* = 0.24), and Hugo RAS (–0.40; 95% CI: − 1.00 to + 0.20; *p* = 0.19). Assessment of between-study heterogeneity revealed negligible dispersion (τ = 0; I² = 0%, 95% CI: 0% to 79%).

The ranking remained identical under both the common and random effects models. Hugo RAS achieved the highest P score (0.94), indicating the greatest probability of being the most effective platform in reducing blood loss. The legacy da Vinci Si system ranked second (0.65), followed by da Vinci Xi (0.55) and da Vinci SP (0.48), suggesting that the established da Vinci platforms cluster within a moderate effectiveness range. In contrast, the investigational systems performed less favorably: MP1000 and KangDuo recorded substantially lower P scores of 0.21 and 0.17, respectively (Fig. 5B).

#### R.E.N.A.L. nephrometry score

**Fig. 6:**
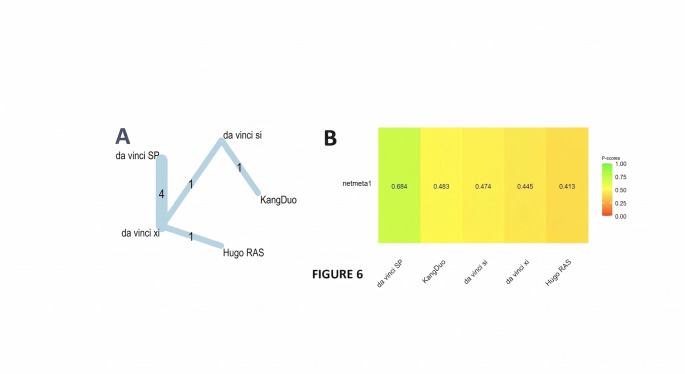
(**A**) Graph network meta-analysis for the effect of robotic surgical platforms on R.E.N.A.L nephrometry score. (**B**) Bar plot showing the platform ranking on R.E.N.A.L nephrometry score

A frequentist, graph-theoretical network meta-analysis was conducted to compare the impact of five robotic surgical platforms on R.E.N.A.L. nephrometry scores: da Vinci Si (reference), da Vinci SP, da Vinci Xi, Hugo RAS, and KangDuo. The analysis synthesized data from 7 trials, incorporating 7 direct comparisons across 4 distinct study designs (Fig. 6A).

Under both the common-effects and random-effects models, no platform demonstrated a statistically significant difference in renal score compared to da Vinci Si. In the random-effects model, effect estimates ranged from an SMD of 0.13 for da Vinci SP (95% CI: − 0.78 to 0.51; *p* = 0.69) to + 0.06 for Hugo RAS (95% CI: − 0.87 to 1.00; *p* = 0.89). Both da Vinci Xi and KangDuo showed null effects (SMD = 0.00), with corresponding confidence intervals spanning − 0.55 to + 0.55 and − 0.64 to + 0.64, respectively (*p* = 1.00 for both).

The network exhibited moderate heterogeneity (τ² = 0.066; τ = 0.26), with an I² of approximately 61% (95% CI: 0% to 87%), suggesting that a meaningful portion of the observed variance may reflect between-study differences.

R.E.N.A.L nephrometry scores: Under the common-effects model, da Vinci SP ranked highest with a P-score of 0.85, suggesting the greatest likelihood of being the most effective system. The remaining platforms were tightly clustered at lower ranks: KangDuo (0.45), da Vinci Si (0.42), da Vinci Xi (0.41), and Hugo RAS (0.37).

When accounting for between-study variability through the random-effects model, the ranking spread narrowed. The P-score for da Vinci SP decreased to 0.68, while the other platforms converged within a narrower range: KangDuo (0.48), Xi (0.44), Si (0.44), and Hugo RAS (0.41) (Fig. 6B). This compression of scores reflects the moderate heterogeneity in the network (τ ≈ 0.26).

### Complications and safety outcomes

Reporting of perioperative complications varied across studies and was not sufficiently consistent to permit formal quantitative synthesis.

Across the included studies, overall complication rates were generally low, and major complications (Clavien–Dindo grade ≥ III) were uncommon when reported. Conversion to open surgery or radical nephrectomy occurred rarely, and transfusion rates were low across most series.

Although the available data suggest comparable safety profiles across robotic platforms, the limited number of comparative studies and heterogeneous reporting prevent definitive conclusions regarding differences in complication rates between systems.

### Quality assessment of studies

**Fig. 7:**
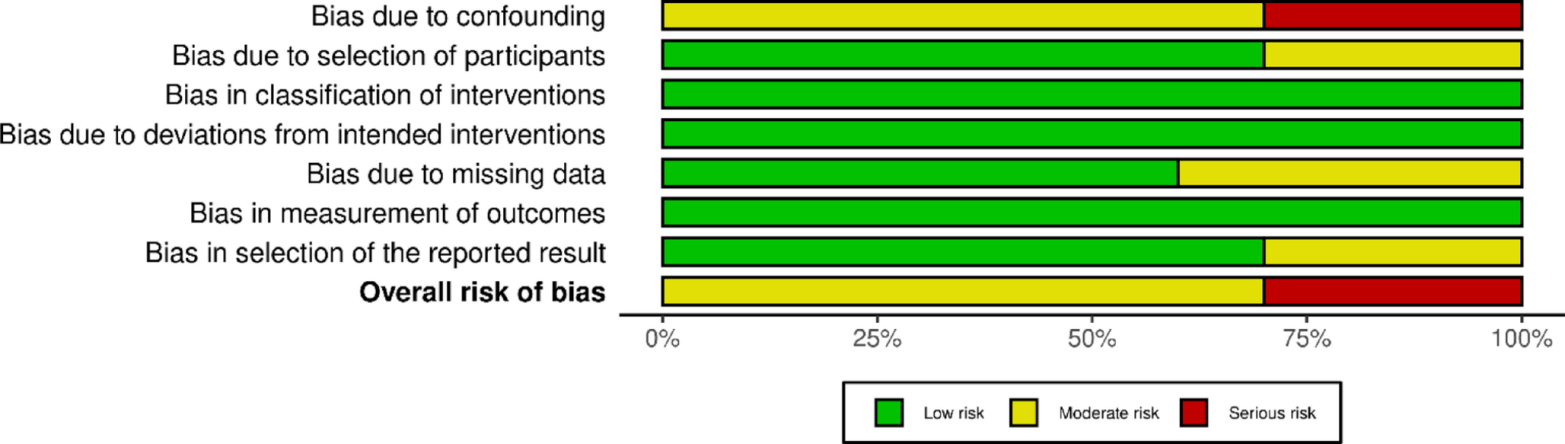
Bar plot of the quality assessment of risk of bias in the included trials using ROBINS-I

The quality assessment included 10 studies. According to Fig. 7, All studies had a low risk of bias concerning intervention classification, outcome measurement and deviations from intended interventions. Biases concerning participant selection, missing data and reported result selection were deemed to be low risk in most studies (6 studies). The most significant concerns were found in El-Asmar (2021), Chen (2023) and Palacios (2022) as they were rated with a high risk of bias due to significant problems with confounding variables. Although the overall confidence in the findings is good, the serious flaws in these three studies introduce certainty and suggest the results should be interpreted cautiously. Further details regarding the assessment are available in Supplementary Figure S1.

## Discussion

To study the outcomes of different robotic platforms used for RAPN, a systematic review and meta-analysis was conducted. To the best of our knowledge, this review represents the first of its kind, addressing a critical gap in current literature and presents several key findings. Thirteen studies were included, involving 2,450 patients and comparing seven robotic platforms, with the largest contributions coming from USA and China.

The first pioneering robotic platform, introduced in the early 2000s, was the da Vinci Surgical System (Intuitive Surgical, Mountain View, CA, USA), which remains the current market leader. Despite its acknowledged advantages, the widespread adoption of this system has been limited by high costs [12, 13]. Among the emerging alternatives, the Hugo RAS System has gained attention as one of the most comprehensive and promising options, particularly in minimally invasive urology [13]. This modular platform features an open console, high-definition monitor, and “pistol-like” hand controllers, offering enhanced communication with operating room staff and facilitating adaptation for experienced laparoscopic surgeons. However, the system still lacks integration of multimodal imaging sources such as ultrasound or 3D reconstructions [14]. Developed by Medtronic (Minneapolis, MN), Hugo RAS was first demonstrated in 2019 with a robotic-assisted radical prostatectomy (RARP) on a cadaver [16]. It received European approval in 2022 and has since emerged as a strong competitor to the da Vinci platform [15]. García Rojo et al. conducted a study comparing the Hugo RAS and da Vinci Xi systems. With no statistically significant differences between the groups in baseline patient characteristics. Docking time was significantly shorter with the da Vinci Xi, while console time was comparable between the two platforms. Differences in unclamp rate, estimated blood loss, and warm ischemia time were not statistically significant. These findings suggest that both platforms allow experienced surgeons to achieve similar surgical performance, potentially leading to equivalent clinical outcomes [14].

Hinotori is a novel robot-assisted surgical system developed in Japan by Medicaroid Corporation (Kobe, Japan). It is the first surgical robot system created in Japan and was also the first worldwide to receive national regulatory approval, granted on August 8, 2020. Following this approval, it entered clinical practice for radical prostatectomy in prostate cancer patients [17, 18]. Since then, its approved indications have expanded in urology. As of April 2022, Hinotori has been approved for use in a many urologic oncologic procedures in Japan, including prostatectomy, partial nephrectomy, radical nephrectomy, cystectomy, and adrenalectomy [18]. In a propensity score–matched study, Motoyama et al. compared the Hinotori system with the da Vinci platform and found no statistically significant differences in major perioperative outcomes, including operative time, console time, warm ischemia time, and estimated blood loss. These findings suggest that RAPN performed with the Hinotori platform provides comparable perioperative outcomes to those achieved with the da Vinci system, even in patients with complex tumors [19]. In contrast, our study demonstrated that Hinotori was associated with a shorter warm ischemia time compared to the da Vinci platform.

Other robotic platforms included in our study were KangDuo and MP1000. The KangDuo-Surgical Robot System is a newly developed platform from China [20]. In a study by Li et al., no significant differences were observed between KangDuo and the da Vinci platform, indicating that KangDuo is not inferior to da Vinci in terms of efficacy or safety. Furthermore, warm ischemia time was similar between the two systems. However, both docking time and suture time per stitch were significantly longer with KangDuo, which may be attributed to the learning curve faced by nurses and surgeons when adopting the new equipment. Notably, docking time showed a significant reduction across all surgeries with KangDuo [21]. MP1000 is a multi-port surgical robot developed by Shenzhen Edge Medical Co., Ltd. (Shenzhen, China). It was approved by the NMPA and entered the market in December 2022 [22]. Surgeons experienced with the da Vinci Si reported that the components and controls of the MP1000 are highly similar, facilitating an easy transition with a relatively smooth learning curve. Moreover, the cost of using MP1000 is estimated to be more than 50% lower compared to the da Vinci platform. In a study by Gao et al., installation time, operative time, and estimated blood loss were found to be comparable between the two systems [23].

The da Vinci SP platform (Intuitive Surgical, Sunnyvale, CA), developed specifically for single-port access, was approved by the United States Food and Drug Administration in 2014 [24, 26]. In a comparative study between the da Vinci SP and the da Vinci multiport systems, ischemia time was found to be significantly longer with the SP. However, no statistically significant differences were observed in overall perioperative outcomes or positive surgical margin rates between them [25].

In our analysis, the da Vinci SP demonstrated the shortest length of hospital stay, whereas the Hugo RAS was associated with the longest. Regarding operative time, the da Vinci SP also had the shortest duration, followed by the da Vinci Xi, while the MP1000 showed the longest operative time. For ischemia time, the da Vinci Xi and Hugo RAS exhibited the shortest durations. However, these findings were not consistent under random-effects models and should be interpreted cautiously.

Although some platforms demonstrated favorable trends under fixed-effects models, these differences were not maintained under random-effects models, indicating that the observed effects may be driven by study-level variability rather than true differences between platforms.

In this network meta-analysis, differences between robotic platforms were generally modest and inconsistent across outcomes. While common-effects models suggested shorter operative and ischemia times for certain newer systems, these differences lost statistical significance once between-study heterogeneity was taken into account.

This finding highlights the substantial variability across the included studies and suggests that observed differences may reflect study-level factors rather than clear superiority of a particular robotic platform.

Substantial heterogeneity was observed for several outcomes, particularly operative time and ischemia time, where I² values exceeded 80%. This variability likely reflects differences in surgeon experience, tumor complexity, patient selection, surgical approach (transperitoneal versus retroperitoneal), and institutional practices across the included studies.

Interpretation of apparent performance differences between robotic platforms should consider potential temporal confounding. Newer systems such as da Vinci Xi, SP, and Hugo RAS were introduced after significant experience with earlier platforms had already been accumulated. Improvements in perioperative outcomes may therefore reflect increasing surgeon experience, refinements in surgical technique, and improvements in perioperative care rather than intrinsic technological advantages of newer robotic systems.

P-score rankings should be interpreted with caution, particularly in the presence of high heterogeneity and overlapping confidence intervals, as they may exaggerate small and clinically insignificant differences.

### Limitations

This study is subject to several important limitations that should be considered when interpreting the findings. The overall strength of evidence is limited by the predominance of observational studies and relatively small sample sizes across included reports. Substantial heterogeneity was observed for key outcomes, likely reflecting differences in surgeon experience, tumor complexity, surgical approach, and institutional practices, which reduces the reliability of pooled estimates. In addition, many studies represent early experiences with emerging robotic platforms, introducing potential learning-curve and early adoption bias, as these technologies are often implemented in high-volume centers by experienced surgeons.

Follow-up duration was generally short, and important long-term outcomes, including oncologic control and renal functional preservation, were inconsistently reported. Several clinically relevant endpoints, such as complications and oncologic outcomes, could not be quantitatively synthesized due to heterogeneous definitions and reporting practices, and were therefore assessed descriptively. Furthermore, the lack of standardized reporting across studies limited the ability to perform subgroup or meta-regression analyses, particularly for factors such as tumor complexity and surgeon expertise.

Tumor complexity represents a major determinant of perioperative outcomes in RAPN. Although R.E.N.A.L. nephrometry scores were reported in several included studies, reporting was heterogeneous and incomplete, preventing formal stratified analyses or meta-regression based on tumor complexity. Consequently, differences observed between robotic platforms may partly reflect variation in case mix rather than intrinsic platform-related performance.

Finally, cost-effectiveness data were largely unavailable, despite their critical importance in evaluating the broader adoption and sustainability of emerging robotic platforms in clinical practice. Furthermore, the absence of cost-effectiveness data highlights an important gap in the available literature. Also, industry-sponsored studies on comparisons between robotic systems may introduce potential biases, as they often favor the sponsor’s platform, limiting the generalizability of our findings. Despite these limitations, our study provides valuable insights into the current and emerging robotic platforms for RAPN and lays the groundwork for future research and clinical practice.

## Conclusion

This systematic review and network meta-analysis shows that newer robotic platforms produce perioperative results that are similar to those of the Da Vinci system. Although certain platforms appeared to perform better in terms of operative time and ischemia time, these differences did not hold up under random-effects models and were overshadowed by considerable heterogeneity across studies.

The available evidence is still preliminary, and it is not possible to conclude that any one platform is superior to another. Larger, well-designed comparative trials with extended follow-up and formal cost-effectiveness evaluations are needed to clarify the role of these emerging technologies in clinical practice.

## Electronic Supplementary Material

Below is the link to the electronic supplementary material.

Supplementary Material 1

Supplementary Material 2: Traffic light plot of the quality assessment of risk of bias in the included trials using ROBINS-I.

## Data Availability

The datasets generated and analyzed during the current study are available from the corresponding author on request.

## Declarations

### AI use

No writing assistance was aided in the manuscript’s preparationor AI use.
