# Supplementary material for: Emerging robotic platforms in partial nephrectomy: a comparative systematic review and network meta-analysis
Source: J Robot Surg. 2026 May 21;20(1):518. doi: 10.1007/s11701-026-03467-6 (PMC13190433; doi:10.1007/s11701-026-03467-6)
Supplement: Supplementary file 1 — Supplementary Material 1 [file 11701_2026_3467_MOESM1_ESM.docx]

Table S.1 Search Strategy of the Systematic Review and Network Meta-Analysis

| Step Number | Search Query |
| --- | --- |
| #1 | ( ( "minimally invasive partial nephrectomy" OR "robotic-assisted partial nephrectomy" OR "RAPN" OR "robot-assisted partial nephrectomy" OR "partial nephrectomy" ) ) |
| #2 | ( ( ( ( "robotic platform" AND ( "Emerging" OR "novel " OR "new " ) ) OR "the Da Vinci" OR "Avatera" OR "Hinotori" OR "Revo-I" OR "Senhance" OR "Versius" OR "KD-SR-01TM" OR "HUGO" OR "MP1000" OR "Carina™") ) ) |
| #3 | 1 AND 2 |

Table S.2 GRADE Summary of Findings for the Network Meta-Analysis

| Outcome | No. of Studies (Patients) | Risk of Bias | Inconsistency | Indirectness | Imprecision | Publication Bias | Overall Certainty | Effect (95% CI) |
| --- | --- | --- | --- | --- | --- | --- | --- | --- |
| Length of Stay | 9 (1466) | Serious | Serious | Not Serious | Serious | Undetected | **Very Low (⊕○○○)** | There are no statistically significant differences between any platform and da Vinci Si. |
| Operation Time | 8 (1371) | Serious | Serious | Not Serious | Not Serious | Undetected | **Low (⊕⊕○○)** | SMD -0.63 (-0.87 to -0.40) for SP vs Si.  SMD -0.52 (-0.67 to -0.38) for Xi vs Si. |
| Ischemia Time | 10 (1520) | Serious | Serious | Not Serious | Serious | Undetected | **Low (⊕⊕○○)** | SMD -0.88 (-1.45 to -0.30) for Hugo vs Si.  SMD -0.58 (-0.73 to -0.44) for Xi vs Si. |
| Blood Loss | 9 (1073) | Serious | Not Serious | Not Serious | Serious | Undetected | **Very Low (⊕○○○)** | No statistically significant differences between any platform and da Vinci Si. (e.g., Hugo RAS SMD -0.40, 95% CI: -1.00 to +0.20). |
| R.E.N.A.L Nephrometry Score | 7 (983) | Serious | Serious | Not Serious | Serious | Undetected | **Very Low (⊕○○○)** | There are no statistically significant differences between any platform and da Vinci Si. |
|  |  |  |  |  |  |  |  |  |
