# Supplementary figures and images for: Emerging robotic platforms in partial nephrectomy: a comparative systematic review and network meta-analysis
Source: J Robot Surg. 2026 May 21;20(1):518. doi: 10.1007/s11701-026-03467-6 (PMC13190433; doi:10.1007/s11701-026-03467-6)

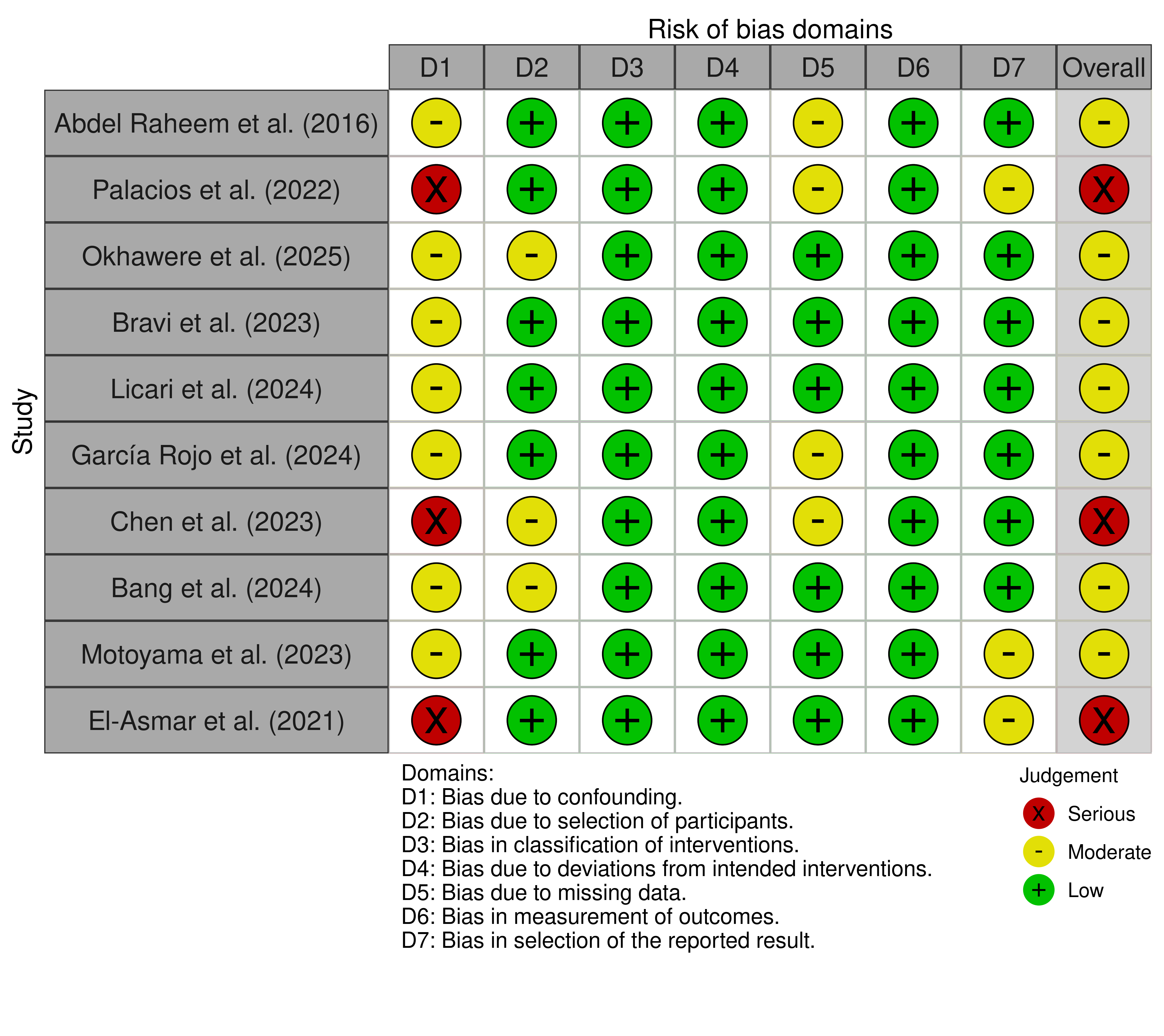

Supplement: Supplementary file 2 — Supplementary Material 2: Traffic light plot of the quality assessment of risk of bias in the included trials using ROBINS-I. [file 11701_2026_3467_MOESM2_ESM.png]
